# New algorithm for OHSS prevention

**DOI:** 10.1186/1477-7827-9-147

**Published:** 2011-11-03

**Authors:** Evangelos G Papanikolaou, Peter Humaidan, Nikos Polyzos, Sofia Kalantaridou, Sahar Kol, Claudio Benadiva, Herman Tournaye, Basil Tarlatzis

**Affiliations:** 1 Human Reproduction & Genetics Foundation, Adrianoupoleos 6, 55133 Kalamaria, Thessaloniki, Greece; 2 The Fertility Clinic Odense University Hospital (OUH) Boulevard 29, entrance 55 5000 Odense C, Denmark; 3 Centrum voor Reproductieve Geneeskunde, UZ Brussel, Flemish Free university of Brussels, Belgium; 4 Department of Obstetrics and Gynecology, University of Ioannina, Greece; 5 Department of Obstetrics and Gynecology, IVF Unit, Rambam Medical Center, Haifa, Israel; 6 Center for Advanced Reproductive Services, University of Connecticut School of Medicine, Department of Obstetrics and Gynecology, Farmington, Connecticut, USA; 7 Assisted Reproduction Unit, 1st Department of Obstetrics and Gynecology, Aristotle University of Thessaloniki, Greece

## Abstract

Ovarian hyperstimulation syndrome (OHSS) still remains a life-threatening complication of in vitro fertilization treatment (IVF), keeping patients and especially those, who previously experienced OHSS, from attempting infertility treatment and childbearing. The recent implementation of four new modalities: the GnRH antagonist protocol, GnRH agonist (GnRHa) triggering of ovulation, blastocyst transfer and embryo/oocyte vitrification, renders feasible the elimination of OHSS in connection with ovarian hyperstimulation for IVF treatment. The proposed current algorithm is based on the number of follicles developed after ovarian stimulation, setting a cut-off level at the development of 18 or more follicles. Further, fulfilling this criterion, the algorithm is based on four decision-making points: the final day of patient work-up, the day of triggering final oocyte maturation, day-1 post oocyte pick-up (OPU) and day-5 post OPU.

If the physician decides to administer hCG for final oocyte maturation regardless the type of analogue used, he has the option on day-1 to either freeze all embryos or to proceed to day-5. On this day, based on the clinical condition of the patient, a decision should be made to either transfer a single blastocyst or to vitrify all blastocysts available. However, this strategy will not guarantee an OHSS free luteal phase especially if a pregnancy occurs. If the physician decides to trigger ovulation with GnRHa, feasible only with the antagonist protocol, embryos can be cultured until day-5. On this day a transfer can be performed with no risk of OHSS and spare blastocysts may be vitrified. Alternatively, on day-1 or day-2 post OPU, all embryos could be frozen.

Hopefully, in a near future, GnRHa triggering and vitrification of oocytes will become everyday practice. Only the combined use of a GnRH antagonist protocol with GnRHa triggering and subsequent single blastocyst transfer or embryo/oocyte freezing will completely abolish the risk of OHSS after ovarian hyperstimulation.

## Background

The most feared complication of IVF-related ovarian stimulation for the patient as well as the doctor is the development of ovarian hyperstimulation syndrome (OHSS) [1]; a syndrome, which in its severe form leads to hospitalization and in the worst case scenario fatal complications. The incidence of clinically significant OHSS is 2-3%, however, milder forms of OHSS might develop in up to 20-30% of all IVF patients [2].

The basis for OHSS development is the development of multiple follicles. Once this criterion is fulfilled, the second factor needed for the development of the severe form of the disease is either the exogenous administration of HCG for final oocyte maturation - as is the current practice - or the establishment of a pregnancy and the production of endogenous HCG from the implanting embryo [3]. Therefore, two types of OHSS have been identified: the early onset OHSS which is self-limited in case no pregnancy occurs, and the late onset OHSS which develops ten days or more after the egg retrieval [4]. In contrast to the early OHSS the late onset OHSS is poorly correlated to the ovarian response after stimulation.

All late onset OHSS cases are related to pregnancy and these cases often require hospitalization. Unfortunately these late OHSS cases render the prediction of OHSS a difficult task [5] and the methods used to predict the condition prior to stimulation have been shown having limited success [6].

The protocol of choice for potential high-responder patients prone to develop OHSS should be the GnRH antagonist protocol, as it has been shown to decrease the incidence of OHSS significantly [7,8]. Furthermore, it allows the utilization of a GnRHa to induce final oocyte maturation, which has recently regained interest. The pooled evidence shows that by triggering with GnRHa in patients co-treated with a GnRH antagonist protocol, not only is OHSS minimized, but also this concept allows embryo transfer in the hyper-responding patient with a reproductive outcome comparable to that seen after hCG triggering as long as adequate luteal support can be achieved [9].

At the same time, the development of the vitrification procedure has improved the embryo survival rate as compared to the classical method of slow freezing [10]. Thus, considering the different modalities that the physician has available, we below propose an algorithm for OHSS high-risk patients. The algorithm may easily be applied according to the preferences of the doctor and his patient and refers to both GnRH-analogues, GnRH agonist as well as GnRH antagonist.

## Algorithm

**Figure 1:**
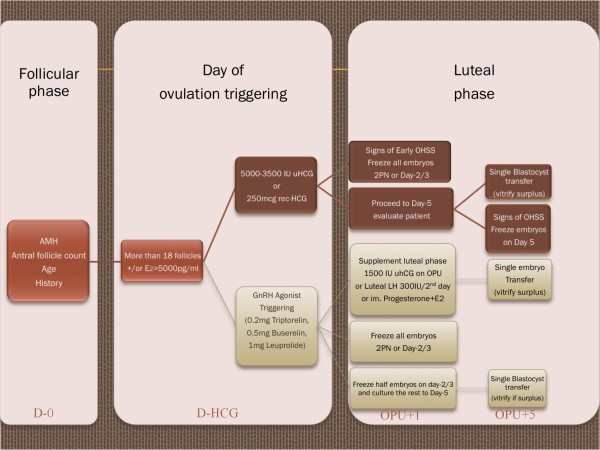
**New proposed algorithm for OHSS prevention and treatment**. The upper pathway (traditional) applies to both the GnRH agonist and GnRH antagonist protocol. The lower pathway (innovative) applies only to the GnRH antagonist protocol.

The algorithm is based on two decision making time periods: the follicular phase and the luteal phase; and four time-points: the final day of patient work-up, the day of ovulation triggering, day-1 post-OPU and day-5 post-OPU (Figure 1). However, the OHSS reducing strategy obviously already starts when the physician evaluates the patient's ovarian reserve and thus the risk for hyperstimulation prior to stimulation [6].

### Final day of patient work-up (Decision point 1)

A mandatory step in the ovarian stimulation for IVF is firstly the gonadotropin dose and secondly the protocol. Regarding the prediction of hyper-response, recent evidence suggests the superiority of AMH over antral follicle count [11,12]. In a prospective cohort of 262 IVF cycles with 8% moderate and severe OHSS, Lee et al showed that an AMH cut-off value of 3.36 ng/mL gave a sensitivity of 90.5% and a specificity of 81.3% to predict OHSS [11,12]. Regarding the antral follicle count, Kwee et al have shown that a cut off level of > 14 antral follicles gave the highest sensitivity (82%) and specificity (89%) and also the highest accuracy. With a prevalence of 15% for high response (defined as > 20 oocytes in an IVF treatment), the accuracy was 88% [13]. According to these thresholds, a woman considered to be at high risk of developing OHSS should be stimulated with low doses of gonadotropins and preferably co-treated with a GnRH antagonist. Nevertheless, individualization of doses and protocol utilized depends on the clinical experience of the treating physician.

Although we use certain cut-off values and our clinical experience to avoid hyperstimulation, we many times fail and the patients ends-up with an excessive ovarian response. As mentioned, Papanikolaou and colleagues have shown that the first prerequisite for OHSS development is the development of multiple follicles and specifically more than 18 follicles with a diameter above 11 mm and/or above 5000 pg/ml Estradiol [2,14]. Thus, from the late follicular phase and onwards, it becomes clear which patient is potentially at high-risk of developing OHSS. Therefore, a modification of the ovarian stimulation should be applied, either decreasing the dose of gonadotropins or administering the triggering bolus of hCG earlier (when three follicles reach the size of 17 mm). In GnRH antagonist protocols, this has been shown to be more efficient compared to later administration at a larger follicular size [15,16]. Both these actions intend to induce the regression of small and medium sized follicles initially recruited, which will decrease the risk of OHSS.

### Day of Triggering final oocyte maturation (Decision 2)

Once the patient has met the criterion set above, the clinician has two options for triggering ovulation: either to administer a lower dose of HCG (250 mcg rec-HCG or 3,300 to 5000 IU urinary hCG), which is the traditional way, applicable in GnRH agonist as well as GnRH antagonist protocols [16]; or to administer GnRHa for final oocyte maturation (0.2 mg Triptorelin or 0.5 mg Buserelin or 1 mg Leuprolide), the innovative way, applicable only in the Antagonist protocol [17].

#### (i) Traditional way with HCG-triggering

hCG for triggering of final oocyte maturation has been the gold standard treatment for decades, clinically approved and highly efficient. This triggering concept is the only one to be applied in patients co-treated with a long GnRH agonist protocol. However, OHSS may still occur despite administration of lower doses of hCG instead of the standard 10,000 IU dose [18]. Specifically in high-risk patients the OHSS incidence might reach up to 30% in case of embryo transfer and the achievement of a pregnancy [19]. On the other hand the presence of exogenous HCG for 8 days in circulation during the luteal phase [18] secures the appropriate function of the corpora lutea and hence the implantation of the transferred embryo.

#### (ii) Innovative way with GnRH Agonist triggering

the newer option, using GnRHa triggering, minimizes the risk of OHSS and secures the appropriate maturation of oocytes. The oocyte pick-up after GnRHa triggering should be performed within 34-35 hours. However, GnRHa triggering is possible only when using a GnRH antagonist protocol and requires modified luteal support in order to be as efficient as hCG triggering [19-21]. The complete eradication of OHSS has made the GnRHa triggering concept the protocol of choice in oocyte donation cycles [22]. In parallel, in IVF with fresh embryo transfer, a new meta-analysis indicates that if luteal support is modified with either a bolus of hCG on the day of OPU, alternate doses of rec-LH, or intense luteal support with intramuscular progesterone and estradiol patches, the delivery rate is comparable to that seen after hCG triggering [9].

### Day-1 in luteal phase post-OPU (Decision 3)

On the first day after the oocyte retrieval, a decision should be made whether to proceed with luteal phase support and subsequently embryo transfer or to freeze all embryos. On this day, the physician will have performed an ultrasound measuring the quantity of fluid in the pouch of Douglas and the ovarian volume; he should also assess the physical condition of the patient i.e. the degree of pain, discomfort, use of painkillers, and breathing difficulty [15]. In addition, the number of fertilized oocytes will be known. Taking these facts into account and adding the efficacy of the cryopreservation program of the unit, a decision can be made whether to proceed with the embryo transfer or to freeze all embryos.

#### (i) Traditional way after HCG-triggering

If the patient on the planned day of triggering already has developed signs or symptoms of early OHSS it is advisable to freeze all embryos at the 2PN stage or at the cleavage stage (day-2/3) and to cancel the embryo transfer. With appropriate counseling the patient will not regard a total freeze as a failure, but rather as a preventive measure, ensuring her health. Importantly, with a good cryo-program her chances of obtaining a pregnancy will not be reduced [23]. Alternatively, given that the patient can tolerate a milder hyperstimulation, a blastocyst transfer can be planned, if her condition does not worsen during the observational period. Nevertheless, the risk of late onset OHSS cannot be excluded with this approach.

#### (ii) Innovative way after GnRH Agonist triggering

As mentioned above, in the new studies focusing on intense luteal support [19] or supplementation with LH-activity [20,21,24] pregnancy rates are comparable to the standard HCG triggering protocol and at the same time OHSS is eliminated. Therefore, a fresh embryo transfer with freezing of surplus embryos might be a more preferred strategy instead of a total freeze. Nevertheless, for those yet not convinced about the reproductive outcome after GnRH agonist triggering followed by modified luteal phase support, a total freeze is still an option.

### Day-5 in luteal phase post-OPU (Decision 4)

Prior to a day-5 transfer, an ultrasound should be performed in combination with blood tests (Hematocrit, WBC, PLT, PT, aPTT, Fibrinogen, D-Dimers, Urea, Creatinine, ALT, AST, gGT, ALP, K, Na, Total Protein, Albumin) to identify patients at high risk of developing severe late onset OHSS. If the evaluation is reassuring a single blastocyst transfer can be performed, followed by vitrification of spare blastocysts [25]. If a patient is considered at high risk of developing OHSS - as might often be the case if hCG triggering has been utilized - the embryo transfer should be cancelled. For units working with slow freezing only or having low blastocyst development rates, an alternative option is to freeze half of the embryos at the cleavage stage and allow the rest to develop into blastocysts. If the condition of the patient permits it, a blastocyst transfer is performed and surplus embryos are cryopreserved.

#### (i) Traditional way after hCG-triggering

on day-5 if the patient confirms her good physical condition and blood tests and/or ultrasound examination are reassuring, a single blastocyst transfer should be performed. Possible supernumerary blastocysts should be vitrified. Importantly, the patient has to be informed that the risk of late OHSS still exists. In contrast, if the patient has developed signs of early onset OHSS, all blastocysts should be cryopreserved.

#### (ii) Innovative way after GnRH Agonist triggering

usually no signs of early OHSS will be present and hence the transfer of a single or even two blastocysts in older patients will not increase the risk of late onset OHSS [9]. Supernumerary blastocysts can be vitrified for future use.

## Competing interests

The authors declare that they have no competing interests.

## Authors' contributions

EGP conceived that concept and wrote the manuscript, PH wrote the manuscript, NPP revised the manuscript, SK revised the manuscript, SK revised the manuscript, HT revised the manuscript, CB wrote the manuscript, BT revised the manuscript. All authors read and approved the final manuscript.
